# Transcriptomic Analysis of Gene Expression Patterns in the Cecal Tissue of Liangshan Yanying Chickens and Arbor Acres (AA) Chickens Before 28 Days of Age

**DOI:** 10.3390/ani16030474

**Published:** 2026-02-03

**Authors:** Zengwen Huang, Jing Wang, Chaoyun Yang, Runjin Wang

**Affiliations:** 1 Xichang University, Xichang 615013, China; 2 Shannan City Livestock and Poultry Improved Breed Breeding Center (Shannan City Tibetan Chicken Industry Research Institute), Shannan 856000, China

**Keywords:** chicken, cecal development, transcriptomics, differentially expressed genes, protein–protein interaction

## Abstract

To dissect the molecular mechanisms underlying chicken cecal development, this study used Liangshan Yanying chickens (a local slow-growing breed) and Arbor Acres (AA) chickens (a fast-growing breed) as experimental models. Cecal tissues were collected from healthy chickens at 1, 14, and 28 days of age (*n* = 10 per breed per day of age) to measure cecal length and perform transcriptome sequencing. Through the screening of differentially expressed genes (DEGs), functional enrichment analysis, construction of protein–protein interaction (PPI) networks, and qRT-PCR validation, temporal changes in cecal development between the two breeds were systematically compared. Results showed that cecal length of both breeds increased significantly with age (*p* < 0.05), with significant differences between breeds. A total of 18 high-quality samples were obtained from transcriptome analysis (Q30 ≥ 93%), with a mapping efficiency of 86.2–90.5%. The number of DEGs was highest between 1 and 28 days of age (1844 DEGs in Liangshan Yanying chickens and 1747 DEGs in AA chickens), and the number of inter-breed DEGs reached 2133 at 28 days of age. A total of 70 DEGs with consistent expression trends were identified (22 upregulated and 48 downregulated), which were enriched in Gene Ontology (GO) terms and Kyoto Encyclopedia of Genes and Genomes (KEGG) pathways such as “B cell activation”, “peptide transport”, and “bile acid metabolism”. qRT-PCR validation indicated that the expression trends of genes (e.g., CD79B, IRF4) were highly consistent with sequencing results (R^2^ = 0.91). PPI network analysis suggested that SLC15A1, ACE, and ENPEP were key hub genes, forming a “transport–metabolism” synergistic module. This study reveals the temporal dynamics of chicken cecal development and the molecular basis of inter-breed differences, providing a theoretical foundation for broiler genetic improvement.

## Simple Summary

To explore the molecular mechanisms underlying cecal development differences between slow-growing local and fast-growing commercial chickens, this study used Liangshan Yanying chickens (local breed) and Arbor Acres (AA) chickens (commercial breed) as models. Cecal length was measured and transcriptome sequencing was performed on cecal tissues at 1, 14, and 28 days of age. Results showed that cecal length of both breeds increased significantly with age, with inter-breed differences gradually narrowing. A total of 70 DEGs with consistent expression trends were identified, enriched in immune response, nutrient transport, and bile acid metabolism pathways. Hub genes such as SLC15A1, ACE, and ENPEP formed a “transport–metabolism” synergistic module. This study reveals the temporal dynamics and inter-breed molecular differences in chicken cecal development, providing candidate genes and theoretical basis for broiler intestinal trait improvement.

## 1. Introduction

Poultry meat ranks as the second most consumed meat in China, with its demand share on a steady upward trajectory annually. The broiler industry, bolstered by its core strengths in high-efficiency production and high-quality output, has emerged as a pivotal driver of structural adjustments within the meat industry [1,2]. Currently, this sector has achieved remarkable progress in large-scale farming and steady advancement in industrialization, forming a mature full-chain development model. Furthermore, shifting demographic structures and rising consumption levels have spurred a transformation in poultry egg and meat consumption toward diversification and personalization. Chicken products characterized by high protein and low fat, alongside cost-effective egg products, have gained widespread market favor, which in turn further fuels the expansion of poultry breeding scales [3]. As a specialized component of the poultry intestinal system, the cecum performs multiple crucial functions, including the absorption of nutrients (e.g., short-chain fatty acids, vitamins), microbial fermentation, and immune defense. Consequently, its developmental status directly impacts broiler growth performance and feed conversion efficiency [4,5]. However, the regulatory mechanisms governing broiler cecal development remain poorly understood, posing a critical bottleneck that restricts industrial breakthroughs in improving growth performance and optimizing feed conversion efficiency—an issue that urgently necessitates systematic research for resolution. Chickens exhibit distinct physiological characteristics compared to other animal species, particularly in the phenotypic divergence of cecal development: fast-growing breeds (e.g., AA broilers) undergo earlier cecal development and form more robust structures. Studies have confirmed significant variations in cecal development across different chicken breeds, with these differences closely linked to genetic background [6,7]. Despite this confirmation of genetically associated phenotypic divergence in cecal development among breeds, the molecular regulatory networks underlying cecal development in local breeds (e.g., Liangshan Yan Ying Chicken) remain elusive. Moreover, systematic analysis of the key genes and pathways responsible for cecal development differences between fast-growing and slow-growing breeds is still lacking, which hinders the precision of genetic improvement for chicken intestinal traits.

In recent years, transcriptomics has become an important tool for dissecting the gene regulatory network of intestinal development. Previous studies using temporal transcriptomic analysis have found that chicken cecal development exhibits stage-specific gene expression patterns [6]. Guan et al. confirmed that dietary metabolizable energy levels can regulate intestinal gene expression, thereby affecting cecal development [8]. Therefore, given the important role of the intestine in potential substance metabolism, a deeper exploration of intestinal functional characteristics may be of great significance for providing clues for strategies to promote intestinal homeostasis (e.g., dietary approaches) [9,10,11]. However, research on the molecular mechanisms underlying inter-breed differences in cecal development remains scarce, especially regarding the exploration of molecular characteristics of cecal development in local chicken breeds (e.g., Liangshan Yanying chickens), which is still insufficient. To reveal the molecular regulatory mechanism of chicken cecal development, this study selected Liangshan Yanying chickens (a slow-growing local breed) and AA chickens (a fast-growing breed) as experimental models. By comparing phenotypic differences in cecal tissues between the two breeds from 1 to 28 days of age and analyzing gene expression patterns using transcriptomics, we screened key genes regulating cecal development and dissected the molecular mechanisms of inter-breed differences in cecal development. The results are expected to fill the gap in temporal transcriptomic research on chicken cecal development, provide theoretical support for the precise improvement of broiler intestinal traits and molecular breeding, and hold important practical significance for enhancing broiler breeding efficiency in animal husbandry.

## 2. Materials and Methods

### 2.1. Ethics

All experiments were conducted in accordance with the Animal Care and Use Guidelines of the Animal Care Committee of Xichang University, China. The protocol was approved by the Animal Care Committee of Xichang University (Approval Number: xcc2022003). All efforts were made to minimize animal suffering and reduce the number of animals used.

### 2.2. Animals and Samples

Fertilized eggs of Liangshan Yanying chickens were procured from Yuexi County Xinyi Ecological Breeding and Planting Professional Cooperative (Liangshan Prefecture, Sichuan, China), and Arbor Acres (AA) broiler fertilized eggs from Suining Zhengda Animal Husbandry Co., Ltd. (Suining, China), 200 eggs per breed. All eggs were disinfected, weighed, numbered, and synchronously incubated in a “Fuhui” integrated incubator-hatcher (Wuhan Fuhui Co., Ltd., Wuhan, China). Incubation parameters were set as follows: 37.8 °C and 60% relative humidity (RH) on days 1–18, with egg turning every 2 h; during days 19–21 (hatching period), temperature was adjusted to 37.2 °C and RH raised to 70%. Candling was conducted on days 7, 11, and 19 to remove infertile eggs, dead sperm eggs, and dead embryo eggs, respectively. The hatching rates were 82.5% for Liangshan Yanying chickens (165 chicks/200 eggs) and 88.0% for AA broilers (176 chicks/200 eggs). Post-hatching, 120 healthy chicks of each breed were selected for rearing, with 4 replicate cages (30 chicks per cage) per breed and extra chicks reserved as backups. Chicks were housed in standardized 2 m × 1 m cages under a cage-raising system, with each cage regarded as the experimental unit. Data were corrected at the cage level in subsequent statistical analyses to ensure reliability. Ad libitum feed and water were provided, following a staged feeding program: pre-starter diet for days 1–14 and starter diet for days 15–28. Both diets, supplied by Kunming Bangyun Feed Co., Ltd. (Kunming, China), met the Chinese Feeding Standard for Chickens (NY/T 33-2004 [12]), with detailed nutritional compositions in Supplementary Material Table S1. Litter and feces were cleaned daily, and cages disinfected to maintain hygiene. No routine immunization was administered to ensure consistent experimental conditions. At 1, 14, and 28 days of age, 10 healthy chicks from each breed were randomly selected. Chicks were euthanized by isoflurane inhalation anesthesia followed by jugular vein exsanguination. Under sterile conditions, cecal length was measured, and cecal tissue samples were collected into sterile cryopreservation tubes, quick-frozen in liquid nitrogen, transported to the laboratory, and stored at −80 °C for subsequent analysis.

### 2.3. Measurement of Cecal Length in Liangshan Yanying Chickens and AA Chickens at Different Ages

Ten Liangshan Yanying chickens and ten AA chickens were randomly selected at 1, 14, and 28 days of age, respectively, and euthanized by jugular vein exsanguination. The chicken abdominal cavity was opened under sterile conditions, the cecal tissue was accurately located, and the total cecal length was measured using a vernier caliper. Data were recorded and statistically analyzed to evaluate differences in cecal length between different ages and breeds. Data were analyzed by Two-Way Analysis of Variance (Two-Way ANOVA) using SPSS software, and the results were presented as the mean ± standard error (SE). A *p*-value < 0.05 was considered statistically significant.

### 2.4. RNA Extraction, Library Construction, and Sequencing Analysis of Cecal Tissues from Liangshan Yanying Chickens and AA Chickens

The RNA-seq experiment was designed with a 2 × 3 × 3 factorial setup, consisting of 2 chicken varieties (Liangshan Yanying chickens and AA chickens), 3 age groups (1, 14, and 28 days of age), and 3 biological replicates per group, resulting in a total of 18 initial samples. After quality assessment, 3 samples with a RNA Integrity Number (RIN) lower than 7.0 were excluded, and the final analysis was conducted using 18 high-quality samples. Notably, all samples were individual specimens without pooled processing to ensure the reliability of experimental results. Experimental subjects were selected based on significant differences in cecal length between Liangshan Yanying chickens and AA chickens. Specifically, chickens of the two varieties were collected at three distinct ages (1, 14, and 28 days of age) for subsequent experiments. The key reagents used in the experiment included: Trizol lysis buffer (Life Technologies, Carlsbad, CA, USA, Cat#15596026); DNase I (Thermo Fisher Scientific, Waltham, MA, USA, Cat#AM2222); RNA Clean & Concentrator-5 Kit (Zymo Research, Irvine, CA, USA, Cat#R1015); and NEBNext Ultra II RNA Library Prep Kit for Illumina (Illumina, San Diego, CA, USA, Cat#E7770). Total RNA was extracted from the collected samples using the Trizol Kit (Invitrogen, Carlsbad, CA, USA). Immediately after extraction, the RNA samples were stored at −80 °C to preserve their integrity for subsequent library construction. RNA samples that met the quality standards were further purified to enrich messenger RNA (mRNA) by removing ribosomal RNA (rRNA). Eukaryotic mRNA with polyadenylated (polyA) tails was specifically enriched using Oligo(dT)-coated magnetic beads. Subsequently, the enriched mRNA was fragmented by ultrasonic treatment. Taking the fragmented mRNA as the template, the first strand of complementary DNA (cDNA) was synthesized. Residual RNA was then degraded by RNase H enzyme, followed by the synthesis of the second cDNA strand. The double-stranded cDNA underwent end repair, polyadenylation, and ligation with sequencing adapters. cDNA fragments with an approximate length of 200 bp were selected for polymerase chain reaction (PCR) amplification. After purification of the PCR products, the sequencing library was successfully constructed using the NEBNext Ultra II RNA Library Prep Kit for Illumina. After library construction, high-throughput sequencing was performed on the Illumina HiSeq2500 platform, with the experiment conducted by Gene Denovo Biotechnology Co., Ltd. (Guangzhou, China).

### 2.5. Clean Reads and Quantification of Gene Abundance

To obtain high-quality sequencing reads, quality control was performed on the raw reads generated by sequencing. Reads containing adapter sequences, unknown nucleotides, low-quality bases (quality score ≤ 20), and ribosomal RNA (rRNA) were removed according to established standards. Subsequently, the high-quality clean reads were aligned to the reference genome using HISAT2 (2.2.1) software [13]. The fragments per kilobase of transcript per million mapped reads (FPKM) values were calculated using StringTie (2.2.3) software to quantify gene expression abundance and variation [14]. DESeq2 (1.42.0) software was used to analyze RNA differential expressions between the two groups, and genes with a false discovery rate (FDR) < 0.05 and absolute fold change (|FC|) ≥ 2 were identified as differentially expressed genes (DEGs) [15].

### 2.6. Bioinformatics Analysis of DEGs

Principal component analysis (PCA) was performed using the gmodels package in R (official website: http://www.r-project.org/). Gene Ontology (GO) annotation was conducted for DEGs from different developmental stages (1, 14, and 28 days of age) and different breeds. GO enrichment analysis of DEGs was performed using clusterProfiler (4.18.4) software and GO terms with statistical significance (*p* < 0.05) were screened, indicating that these terms were significantly enriched in DEGs. In addition, Kyoto Encyclopedia of Genes and Genomes (KEGG) pathway enrichment analysis was performed for DEGs.

### 2.7. Data Processing and Analysis

Transcriptomic data were processed and analyzed using R 4.0 software (gmodels, clusterProfiler packages) and SPSS 28.0 (IBM Corp., Armonk, NY, USA). WPS Office was only used for data collation and preliminary table drafting. Data were presented as mean ± standard deviation (SD), and comparisons between the control group and experimental groups were performed using one-way ANOVA. The statistical significance level was set at *p* < 0.05. In addition, data visualization was performed using Origin 2022 and R 4.0.

### 2.8. Real-Time Quantitative PCR

To validate the differentially expressed genes (DEGs) identified by bioinformatics analysis, quantitative real-time PCR (qRT-PCR) was performed on selected DEGs. These genes, including CD79B, IRF4, BLVRA, CLCA1, ALPI, and SLC9A3, were chosen based on functional enrichment analysis results and significant expression differences observed in the preceding RNA-seq data. Primers for the target genes were designed using Premier 5.0 software with reference to the corresponding gene sequences retrieved from the NCBI database, and the detailed primer information is provided in Supplementary Table S2. All primers were synthesized by Sangon Biotech (Shanghai) Co., Ltd. (Shanghai, China) The qRT-PCR experiments were conducted using ArtiCanATM SYBR qPCR Mix (Qingke Biotechnology, Beijing, China) on an ABI StepOnePlus Real-Time PCR System (Life Technologies, USA). The total volume of each reaction system was 20 μL, consisting of 10 μL of SYBR Mix, 0.5 μL of forward primer, 0.5 μL of reverse primer, 2 μL of cDNA template, and 7 μL of double-distilled water (ddH_2_O).The thermocycling program was set as follows: an initial pre-denaturation step at 95 °C for 3 min, followed by 40 cycles of denaturation at 95 °C for 10 s and combined annealing/extension at 60 °C for 30 s. For experimental design, three biological replicates were randomly selected from each RNA-seq sample, covering different varieties and age groups, and each biological replicate was subjected to three technical replicates to ensure the reliability of results. β-actin (GenBank Accession No.: NC_052545.1) was used as the reference gene to normalize the expression levels of target genes, and the relative expression levels of DEGs were calculated using the 2^−△△Ct^ method.

## 3. Results

Determination and Analysis of Cecal Length in Liangshan Yanying Chickens and AA Chickens at Different Ages.

To investigate the growth and developmental characteristics of cecal tissues in Liangshan Yanying Chickens and AA Chickens, a 2 × 3 two-factor experimental design was employed in this study. Ten chicks were randomly selected from each breed at 1, 14, and 28 days of age, respectively. Following humane euthanasia via jugular vein exsanguination, cecal length was accurately measured with a vernier caliper. The collected data were first subjected to normality test using the Shapiro–Wilk method, followed by two-way analysis of variance (two-way ANOVA) coupled with Tukey’s HSD post hoc test for multiple comparisons. The results showed that from day 1 to day 28, the cecal length of both Liangshan Yanying Chickens and AA Chickens exhibited a continuous increasing trend with increasing age, with extremely significant differences observed among different ages within the same breed (F = 1197.49, *p* < 0.001). Detailed analysis results are presented in Tables S3 and S4. Further comparative analysis demonstrated that at the same age, there were also extremely significant differences in cecal length between the two breeds (F = 1054.90, *p* < 0.001), and detailed data and analysis results are shown in Table 1. Notably, an extremely significant interaction effect was observed between breed and age (F = 83.59, *p* < 0.001), with specific patterns as follows: at day 1, the cecal length of Liangshan Yanying Chickens was significantly shorter than that of AA Chickens; the difference between the two breeds narrowed significantly at day 14; at day 28, the cecal length of Liangshan Yanying Chickens remained shorter than that of AA Chickens, but the discrepancy was further diminished. These findings indicate that the cecal development rate of Liangshan Yanying Chickens is relatively slow; however, the developmental gap with AA Chickens gradually diminishes with increasing age, showing a growth characteristic of gradual approximation.

### 3.1. Statistical Analysis of Transcriptome Sequencing Genes in Cecal Tissues of Liangshan Yanying Chickens and AA Chickens at Different Ages

**Figure 1:**
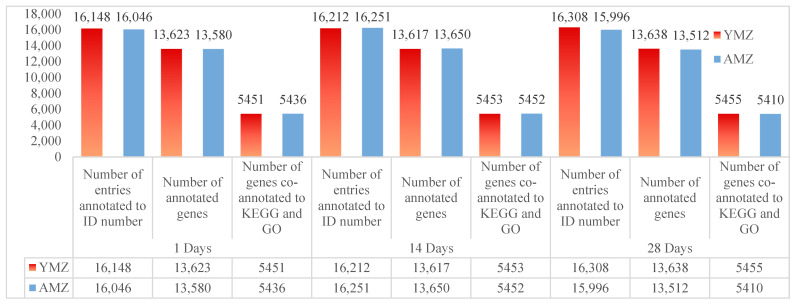
Statistical analysis of transcriptome sequencing genes in cecal tissues of Liangshan Yanying chicken and AA chicken at different ages. Note: YMZ: Represents the cecal tissue of Liangshan Yanying chicken; AMZ: Represents the cecal tissue of AA chicken.

After strict quality control of samples and sequencing procedures, the sequencing data were aligned with the data in the *Gallus gallus* (chicken) database. The FPKM values of each sample were calculated using StringTie software. After screening and analysis, under the condition of FPKM > 0, the number of annotated ID entries in the cecal tissues of Liangshan Yanying chickens at 1, 14, and 28 days of age was 16,148, 16,212, and 16,308, respectively; while the number of annotated ID entries in the cecal tissues of AA chickens at the corresponding ages was 16,046, 16,251, and 15,996, respectively. After filtering and gene annotation of the ID annotation data in cecal tissues of Liangshan Yanying chickens at different ages, the number of expressed genes annotated in the cecal tissues at 1, 14, and 28 days of age was 13,623, 13,617, and 13,638, respectively; the number of expressed genes annotated in the cecal tissues of AA chickens at the corresponding ages was 13,580, 13,650, and 13,512, respectively. Further combined KEGG and GO annotation analysis of expressed genes in cecal tissues of Liangshan Yanying chickens and AA chickens at different ages showed that the number of genes co-annotated to KEGG and GO in the cecal tissues of Liangshan Yanying chickens at 1, 14, and 28 days of age was 5451, 5453, and 5455, respectively; the number of genes co-annotated to KEGG and GO in the cecal tissues of AA chickens at the corresponding ages was 5436, 5452, and 5410, respectively. The above data results are presented in Figure 1.

### 3.2. Analysis of Inter-Sample Relationships in Transcriptome Sequencing Data of Cecal Tissues from Liangshan Yanying Chickens and AA Chickens at Different Ages

**Figure 2:**
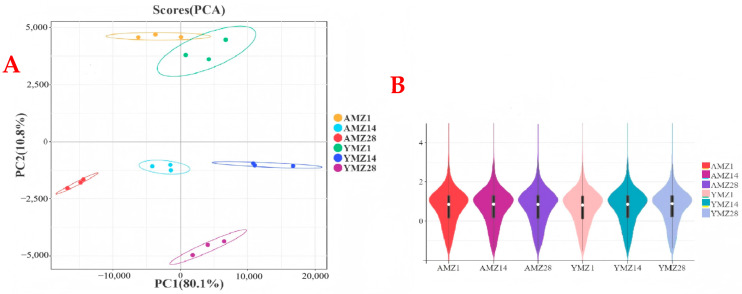
(**A**) PCA of cecal tissue group samples from different growth ages of Liangshan Yangying chicken and AA chicken. (**B**) Violin plot showing the distribution of inter-group differences in sample sequencing data. Note: AMZ1 represents the transcriptome sequencing data of cecal tissue from 1-day-old AA chickens, AMZ14 represents the transcriptome sequencing data of cecal tissue from 14-day-old AA chickens, AMZ28 represents the transcriptome sequencing data of cecal tissue from 28-day-old AA chickens; YMZ1 represents the transcriptome sequencing data of cecal tissue from 1-day-old Liangshan Yanying chickens; YMZ14 represents the transcriptome sequencing data of cecal tissue from 14-day-old Liangshan Yanying chickens; YMZ28 represents the transcriptome sequencing data of cecal tissue from 28-day-old Liangshan Yanying chickens.

To study gene expression in cecal tissues of Liangshan Yanying chickens and AA chickens at different growth ages (1, 14, and 28 days of age) and the results of differential gene expression between breeds (Liangshan Yanying chickens and AA chickens), transcriptome sequencing was performed on cecal tissues of the two breeds at the three ages. After quality control of the sequencing data, PCA results showed that cecal tissue samples of different growth ages (1, 14, and 28 days of age) and different breeds (Liangshan Yanying chickens and AA chickens) exhibited a significant aggregation trend in intra-group scatter plots, indicating high intra-group reproducibility and high consistency of sample data; meanwhile, inter-group scatter plots showed obvious distinguishability (Figure 2A). These findings verify the high reliability of the sequencing data, laying a foundation for subsequent DEG analysis. In Figure 2A, the distribution of samples from different groups can be observed through scatter plots of different colors or shapes. The figure clearly shows the tight aggregation of intra-group samples and significant distinction of inter-group samples, further verifying the validity and accuracy of PCA and providing strong support for biological analysis. In addition, a violin plot was used to show the data distribution and the degree of gene expression dispersion (Figure 2B). The results showed that the median, interquartile range, and gene expression distribution within the same group were similar, indicating high reproducibility of parallel samples within the same group. Both sample correlation analysis and violin plot results indicated that the transcriptomic data were of high quality and suitable for further analysis.

### 3.3. Analysis of DEGs in Cecal Tissues Between Liangshan Yanying Chickens and AA Chickens at Different Ages and Breeds

**Figure 3:**
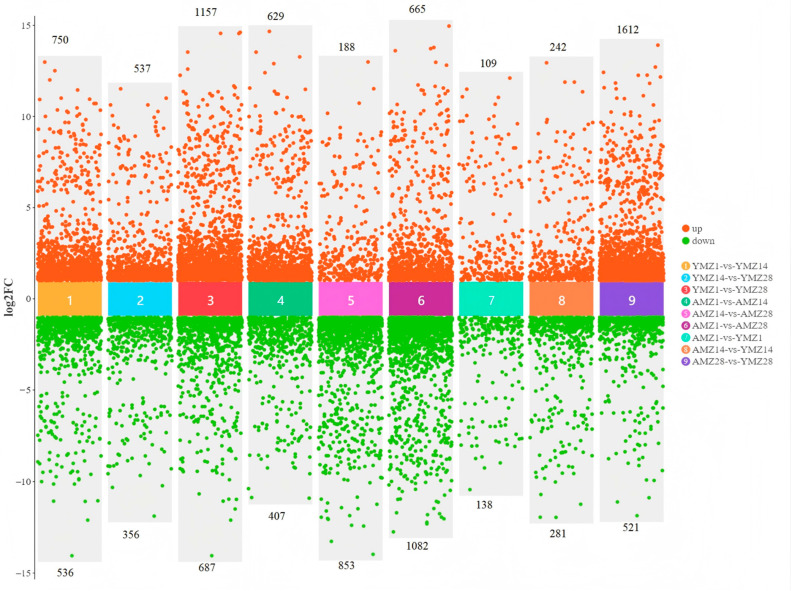
Differentially expressed genes in cecal tissues of Liangshan Yanying chicken and AA chicken across different age groups and breeds.

To deeply study the specific differences in gene expression in cecal tissues between Liangshan Yanying chickens and AA chickens at different developmental stages, gene expression levels were screened with the criteria of |log_2_FC| > 1 and FDR < 0.05. The results showed that between 1 and 14 days of age, a total of 1286 genes were differentially expressed in the cecal tissues of Liangshan Yanying chickens, among which 750 genes were upregulated and 536 genes were downregulated at 1 day of age. Between 14 and 28 days of age, 893 DEGs were identified in the cecal tissues of Liangshan Yanying chickens, with 537 genes upregulated and 356 genes downregulated at 14 days of age. In addition, between 1 and 28 days of age, a total of 1844 DEGs were found in the cecal tissues of Liangshan Yanying chickens, including 1157 upregulated genes and 687 downregulated genes at 1 day of age. For AA chickens, a total of 1036 DEGs were identified between 1 and 14 days of age, with 629 genes upregulated and 407 genes downregulated at 1 day of age. Between 14 and 28 days of age, 1041 DEGs were found in the cecal tissues of AA chickens, among which 188 genes were upregulated and 853 genes were downregulated at 14 days of age. Finally, between 1 and 28 days of age, a total of 1747 DEGs were identified in the cecal tissues of AA chickens, with 665 genes upregulated and 1082 genes downregulated at 1 day of age. Further study on the specific differences in gene expression in cecal tissues between Liangshan Yanying chickens and AA chickens at the same age showed that at 1 day of age, a total of 247 DEGs were found between AA chickens and Liangshan Yanying chickens, among which 109 genes were upregulated and 138 genes were downregulated in AA chickens compared with Liangshan Yanying chickens. At 14 days of age, the total number of DEGs reached 523, with 242 genes upregulated and 281 genes downregulated in AA chickens compared with Liangshan Yanying chickens. By 28 days of age, the total number of DEGs increased significantly to 2133, among which 1612 genes were upregulated and 521 genes were downregulated in AA chickens compared with Liangshan Yanying chickens. The analysis results are shown in Figure 3.

### 3.4. Analysis of Gene Expression Patterns in Cecal Tissues of Liangshan Yanying Chickens and AA Chickens Before 28 Days of Age

**Figure 4:**
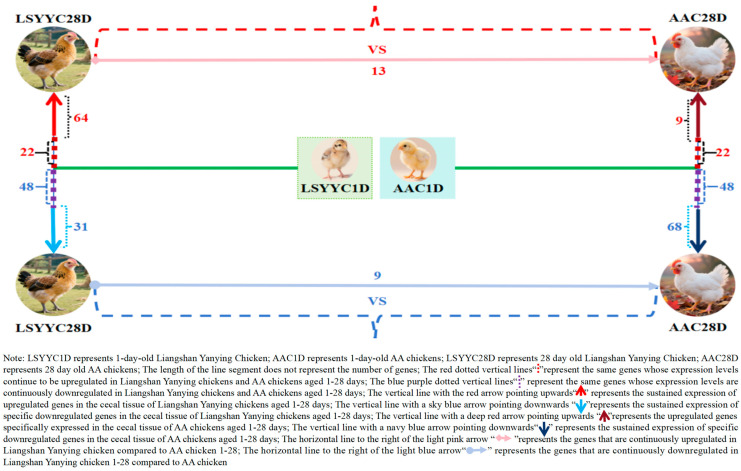
Pattern of gene expression changes in cecal tissue of Liangshan Yanying chicken and AA chicken aged 1–28 days.

To deeply explore the dynamic changes in gene expression in chicken cecal tissues before 28 days of age, trend analysis was performed on DEGs between different growth stages and breeds of Liangshan Yanying chickens and AA chickens. The results showed that from 1 to 28 days of age, the number of genes with continuously upregulated expression in the cecal tissues of Liangshan Yanying chickens was 86, while that in AA chickens was 31. Meanwhile, there were 22 genes with continuously upregulated expression in both breeds. Regarding downregulated genes, from 1 to 28 days of age, the number of genes with continuously downregulated expression in the cecal tissues of Liangshan Yanying chickens was 79, and that in AA chickens was 116. A total of 48 genes showed continuously downregulated expression in both breeds. Further analysis found that at the same age, 13 genes were upregulated and 9 genes were downregulated in Liangshan Yanying chickens compared with AA chickens. These results reveal the dynamic differences in gene expression in cecal tissues between Liangshan Yanying chickens and AA chickens before 28 days of age, providing basic data for dissecting the molecular mechanisms of cecal development in the two breeds. The data visualization results are shown in Figure 4.

### 3.5. Functional Analysis of DEGs in Cecal Tissues of Liangshan Yanying Chickens and AA Chickens Before 28 Days of Age

**Figure 5:**
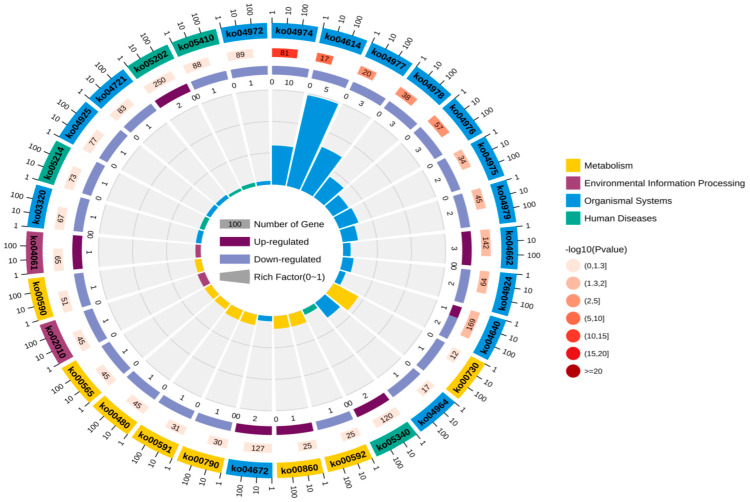
KEGG annotation analysis of continuously differentially expressed genes in Liangshan Yanying chicken and AA chicken aged 1–28 days.

**Figure 6:**
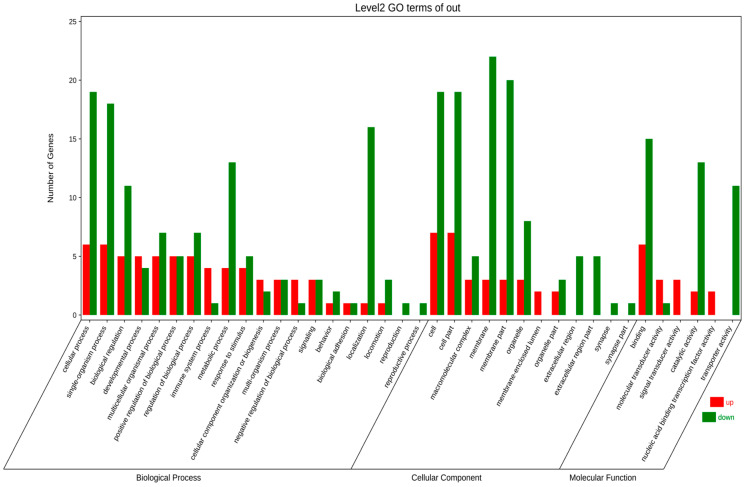
GO annotation analysis of continuously differentially expressed genes in Liangshan Yanying chicken and AA chicken aged 1–28 days.

Through in-depth analysis of cecal tissues from Liangshan Yanying chickens and AA chickens during the 1–28-day growth period, this study identified distinct dynamic changes in gene expression profiles. Specifically, 22 genes exhibited a consistent upregulation trend throughout this growth stage in both chicken breeds. However, only 8 out of these 22 genes were successfully annotated via KEGG and GO enrichment analyses. Concurrently, 48 genes displayed a persistent downregulation pattern in both breeds from day 1 to day 28, among which 24 were effectively annotated using the same enrichment approaches. KEGG pathway enrichment analysis demonstrated that the differentially expressed genes (DEGs) were primarily enriched in four functional categories: “Metabolism”, “Environmental Information Processing”, “Organismal Systems”, and “Human Diseases” (Figure 5). Similarly, GO term enrichment analysis revealed that DEGs were concentrated in three major biological domains: “Biological Process”, “Cellular Component”, and “Molecular Function”. Among these domains, “Biological Process” contained the highest number of enriched terms (20), followed by “Cellular Component” (12 terms), while “Molecular Function” had the fewest (6 terms).Subsequent in-depth analysis indicated that the “membrane”-related pathway harbored the greatest number of enriched genes, all of which exhibited a downregulation trend. In contrast, the “cell”-related pathway contained the largest subset of upregulated genes (Figure 6). Upregulated expression of these genes is hypothesized to play a crucial role in the early growth and development of chickens, whereas downregulated genes also exert essential regulatory effects during this critical period. Gene selection was conducted based on the following criteria: ① significant enrichment in core pathways, including immune response, material metabolism, and nutrient transport; ② stringent differential expression thresholds (|log_2_FC| > 2 and FDR < 0.01) to ensure statistical significance; ③ prior literature evidence supporting associations with intestinal development, immune function, or nutrient absorption (e.g., CD79B participates in B cell activation, IRF4 regulates immune cell development, and SLC9A3 is involved in ion transport); ④ coverage of diverse functional categories to guarantee the representativeness of validation results.

### 3.6. qRT-PCR Validation of DEGs in Cecal Tissues of Liangshan Yanying Chickens and AA Chickens Before 28 Days of Age

**Figure 7:**
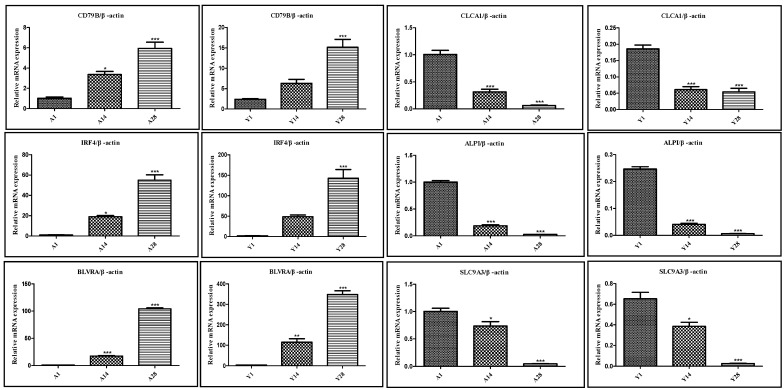
qPCR validation results of genes exhibiting sustained expression changes in Liangshan Yanying chicken and AA chicken from 1 to 28 days post-hatch. Note: * indicates a difference with no statistical significance; ** indicates a statistically significant difference; *** indicates a highly statistically significant difference.

To clarify the research objectives and highlight the research value, based on the results of GO and KEGG enrichment analysis of DEGs and gene function annotation, specific genes (CD79B, IRF4, BLVRA, CLCA1, ALPI, and SLC9A3) with potential effects on the growth and development of chicken cecal tissues were selected for qRT-PCR validation. qRT-PCR was used to verify the RNA-Seq expression data, confirming the reliability of the RNA-Seq results. Specifically, the expression levels of CD79B, IRF4, and BLVRA genes increased significantly with age, while the expression levels of CLCA1, ALPI, and SLC9A3 genes decreased significantly with age. The qRT-PCR results of these genes were highly consistent with the RNA-Seq analysis results, verifying the accuracy and credibility of the analysis results. Detailed measurement results are shown in Figure 7.

### 3.7. PPI Network Analysis of DEGs in Cecal Tissues of Liangshan Yanying Chickens and AA Chickens Before 28 Days of Age

**Figure 8:**
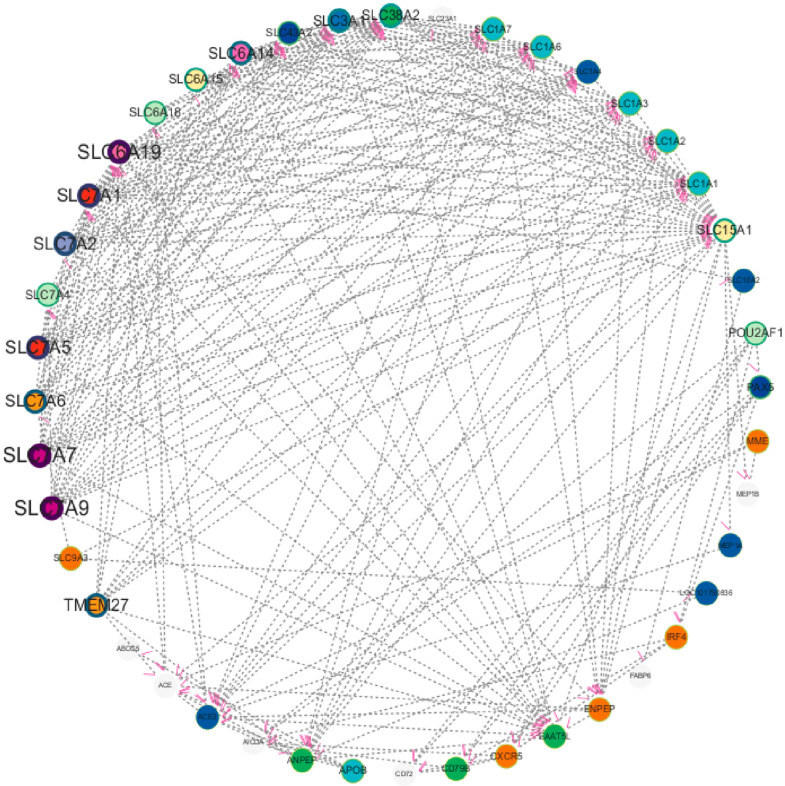
Molecular network interaction of continuously changing gene expression in Liangshan Yanying chicken and AA chicken aged 1–28 days.

To deeply explore the molecular regulatory mechanism during chicken cecal growth and development, this study first screened 32 DEGs using transcriptome sequencing technology and then conducted PPI analysis with these genes as the core. The potential interaction relationships of proteins encoded by these genes were predicted using the String database (https://string-db.org/), and the network was constructed and visually optimized using Cytoscape (3.10.2) software. Finally, a multi-gene PPI network was obtained (Figure 8). To ensure the reliability of the analysis results, the research team strictly screened according to the protein interaction score, removed interaction relationships with low credibility, and retained high-confidence interaction pairs. The results showed that in the screened network, proteins such as SLC15A1, SLC3A1, SLC6A19, ENPEP, MME, ACE, and ANPEP exhibited significant hub characteristics, i.e., they had more direct interaction relationships with other proteins, suggesting that these genes may be in key node positions in the regulatory network. Notably, further analysis found that these highly interactive genes also had close interaction relationships with each other, forming a synergistically regulated molecular module. The PPI network results were not only highly consistent with the DEGs screened by previous transcriptome sequencing, re-verifying the reliability of the transcriptomic data from the perspective of protein interaction, but more importantly, this finding suggests that the above genes may jointly participate in the regulation of chicken cecal growth and development through synergistic effects, providing important network biological evidence for dissecting the molecular mechanism of chicken cecal development.

### 3.8. Biological Function Analysis of DEGs in Cecal Tissues of Liangshan Yanying Chickens and AA Chickens Before 28 Days of Age

**Figure 9:**
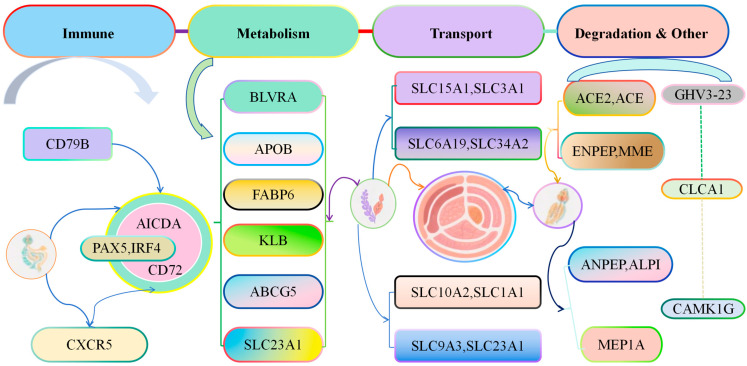
Functional Analysis of Genes with Continuously Changing Expression in the Cecal Tissues of Liangshan Yanying Chickens and AA Chickens aged 1–28 days.

Functional analysis of genes with continuous changes in expression levels in cecal tissues of Liangshan Yanying chickens and AA chickens from 1 to 28 days of age showed that these genes were involved in multiple core physiological processes, including development, activation, and immune response of immune cells (e.g., CD79B, PAX5, IRF4, AICDA, CD72, CXCR5); metabolism and homeostasis regulation of substances (e.g., lipids, bile acids, vitamins, bilirubin) in the body (e.g., BLVRA, APOB, FABP6, KLB, ABCG5, SLC23A1); transmembrane transport of substances such as amino acids, peptides, ions, and bile acids through the solute carrier (SLC) family or transporters (e.g., SLC15A1, SLC3A1, SLC6A19, SLC34A2, SLC10A2, SLC1A1, SLC9A3); degradation or transformation of biomolecules such as peptides, hormones, and lipids (e.g., ACE2, ACE, ENPEP, MME, ANPEP, ALPI, MEP1A); genes with other functions (e.g., IGHV3-23, CLCA1, CAMK1G) (Figure 9). The functions of these genes cover multiple key physiological processes such as immune response, substance metabolism, nutrient absorption, and signal regulation. Their abnormal expression or mutation is not only closely related to diseases such as immune diseases, metabolic syndromes, and tumors, making them important targets for medical research and drug development but also closely related to chicken growth and development and disease prevention.

## 4. Discussion

As an important global poultry species, chickens provide humans with eggs and chicken meat, which are major sources of protein, essential amino acids, and minerals [16]. The intestine, as the core of the digestive system, undertakes the key functions of nutrient digestion and absorption. As a specialized organ of the poultry intestinal system, the cecum has more complex physiological roles, including nutrient absorption, osmotic pressure regulation, vitamin synthesis, microbial fermentation, and immune defense [17,18,19]. In terms of nutritional factors, studies by Guan et al. [20], Hu [21], and Tang et al. [22] have confirmed that dietary metabolizable energy levels regulate cecal development by affecting intestinal length and weight, and high metabolizable energy levels may even inhibit cecal growth. Although this study did not directly involve nutritional intervention, the phenotypic differentiation of cecal development between breeds provides a basis for research on genetic–nutritional interactions. In addition, González-Alvarado et al. found that dietary crude fiber can promote cecal development by stimulating intestinal peristalsis, suggesting that nutritional regulation may indirectly affect cecal phenotype through gene expression [23].

Transcriptomic analysis revealed the gene expression characteristics of cecal development. This study found that the number of DEGs in cecal tissues between the two breeds increased with age, with the most significant differences between 1 and 28 days of age, indicating that cecal gene expression has temporal specificity, which is closely related to the nutritional needs, environmental adaptation, and developmental processes of chickens at different growth stages. Functional enrichment analysis showed that DEGs were mainly involved in core physiological processes such as immune response, substance metabolism, and nutrient absorption. The continuous differential expression of 8 immune-related genes further confirms the functional characteristic of the intestine as a “dual barrier for digestion and immunity” [24,25,26]. The early stage (1–14 days of age) represents a critical period for cecal morphogenesis. During this period, the differentially expressed genes (DEGs) of the two chicken breeds are mainly enriched in pathways related to cell proliferation and basic metabolism (e.g., ribosomal biogenesis and glycolysis pathway). Breed differences are relatively minor, probably because cecal development at this stage focuses on universal morphological construction, which is regulated by species-conserved genes. In the late stage (14–28 days of age), the cecum enters a functional maturation phase. The DEGs then shift to pathways associated with immune response and specific nutrient absorption (e.g., B cell activation and bile acid metabolism), and breed differences expand significantly. This phenomenon is linked to the genetic background divergence between the two breeds: as a fast-growing variety, AA broilers need to rapidly optimize nutrient absorption to support growth; in contrast, Liangshan Yanying chickens prioritize the establishment of intestinal immune homeostasis for better environmental adaptation. Such functional specialization ultimately leads to a sharp surge in the number of DEGs in the late stage.

Regarding key genes and pathways, the solute carrier (SLC) gene family exerts a prominent role [27,28]. As a sodium-dependent transporter, SLC13A1 is pivotal to nutrient absorption [29,30], and hub genes like SLC15A1 identified herein regulate cecal function via a “transport–metabolism” coordinated network—consistent with the conserved substrate transport function of the SLC family in mammals [27,28]. This network comprises two functional modules: a transport module (SLC15A1, SLC3A1, SLC6A19) mediating transmembrane transport of peptides and amino acids, and a metabolic module (ENPEP, ACE, ACE2) involved in degrading peptide hormones and bioactive peptides. Notably, this study is the first to report differential expression of renin-angiotensin system-related genes (ENPEP, ACE, ACE2) in the chicken cecum. Given its role in regulating intestinal mucosal function in rats [10,31], this system likely exerts a conserved function in chicken cecal development, offering new clues for cross-species comparative studies. Moreover, fatty acid-binding protein 6 (FABP6) in the PPAR signaling pathway not only participates in lipid metabolism but also links to intestinal barrier function through differential expression—aligning with prior findings that FABP6 downregulation associates with intestinal barrier dysfunction in broilers [25,32,33].

For breed-specific differences, differential expressions of the bile acid metabolism pathway serve as the core driver of cecal functional divergence between AA broilers and Liangshan Yanying chickens. AA broilers highly express bile acid transport and metabolism genes (e.g., ABCG5, SLC10A2), boosting fat digestion and absorption efficacy to meet the high energy demands of rapid growth. Coupled with their longer cecum and high expression of the SLC-mediated “transport–metabolism” network, AA broilers enable rapid nutrient transport, efficient peptide and amino acid metabolism, and accelerated cecal functional maturation—collectively underpinning their rapid growth and superior feed conversion efficiency. In contrast, Liangshan Yanying chickens show low expression of these pathway and network genes, potentially prolonging nutrient residence in the cecum to facilitate microbial fermentation (for short-chain fatty acid production as energy) and immune-related substance synthesis, adapting to extensive feeding conditions.

Liangshan Yanying chickens also exhibit sustained high expression of immune-related genes (CD79B, PAX5, IRF4), which mediate B cell activation and antibody production. This expression profile enhances intestinal mucosal humoral immunity, improves resistance to environmental pathogens, and mirrors the strong stress resistance phenotype of local breeds—a genetic trait shaped by long-term natural selection. Meanwhile, FABP6 shows breed-specific expression: its low level in AA broilers correlates with rapid intestinal barrier maturation, while its high level in Liangshan Yanying chickens reinforces barrier stability and mitigates external stimulus-induced intestinal damage. Integrated phenotypic and transcriptomic analysis elucidates the age- and breed-specific mechanisms underlying cecal developmental differences between the two breeds, establishing a theoretical basis for broiler molecular breeding. Identified key genes (e.g., SLC15A1, FABP6) serve as candidate targets for intestinal trait improvement, and novel findings (e.g., gluconeogenesis and renin-angiotensin system involvement in cecal function) broaden insights into poultry intestinal physiology.

However, this study has limitations: first, the interaction mechanism between intestinal microbiota and host genes is not fully elucidated, and the specific pathway by which short-chain fatty acids (SCFAs) regulate cecal development via enterocyte proliferation stimulation [34] warrants further investigation; second, the genetic–nutritional synergistic regulatory network requires validation via more intervention experiments. Future studies may integrate CRISPR technology and intestinal microbiota modulation to decipher key gene functions and application values, providing practical guidance for improving broiler breeding efficiency.

## 5. Conclusions

The results of this study show that the cecal length of both Liangshan Yanying chickens and AA chickens increases significantly with age, while the inter-breed difference gradually decreases. This phenomenon reveals the genetic specificity and temporal adaptability of cecal development. Through transcriptomic analysis, 70 genes with co-expression trends were identified in the two breeds, which are mainly involved in physiological processes such as immune response, nutrient absorption, and substance metabolism. Genes such as SLC15A1, ACE, and IRF4 regulate cecal development through a synergistic network and are expected to be candidate genes for improving broiler intestinal traits.

## Figures and Tables

**Table 1:** Comparison of cecal length between Liangshan Yanying chicken and AA chicken at different age Unit: cm *n* = 10.

Day-Old	Breed	
	Liangshan Yanying Chicken	AA Chicken
1 Days	3.76 ± 0.08 ^A^	5.49 ± 0.15 ^A^**
14 Days	5.28 ± 0.08 ^B^	9.72 ± 0.47 ^B^**
28 Days	8.98 ± 0.37 ^C^	14.25 ± 0.92 ^C^**

Note: In the same column, different capital letters indicate extremely significant differences (*p* < 0.01), and the same letter indicates no significant difference; in the peer column, ** indicates extremely significant differences (*p* < 0.01).

## Data Availability

All raw data generated in this study have been deposited in the China National Center for Bioinformation (https://ngdc.cncb.ac.cn/gsa/ (accessed on 30 January 2026)), with the transcriptome data under accession number “PRJCA042510”.

## Supplementary Materials

The following supporting information can be downloaded at: https://www.mdpi.com/article/10.3390/ani16030474/s1, Table S1: List of Q-PCR verification primer sequences for some genes. Table S2: Optimal Nutrient Levels of Pre-starter and Starter Diets for Broiler Chicks During the Starter Phase. Table S3: Normality Test (Shapiro-Wilk Test) of Cecal Length in Liangshan Yanying Chicken and AA Chicken. Table S4: Two-Way Analysis of Variance (Two-Way ANOVA) of Cecal Length in Liangshan Yanying Chicken and AA Chicken of Different Ages.
