# Supplementary material for: Transcriptomic Analysis of Gene Expression Patterns in the Cecal Tissue of Liangshan Yanying Chickens and Arbor Acres (AA) Chickens Before 28 Days of Age
Source: Animals (Basel). 2026 Feb 3;16(3):474. doi: 10.3390/ani16030474 (PMC12896458; doi:10.3390/ani16030474)
Supplement: Supplementary file 1 [file animals-16-00474-s001.zip › Supplementary Table S1.docx]

**Supplementary Table S1 List of Q-PCR verification primer sequences for some genes**

| **Gene Name** | **Sequence（5’-3’）** | **Size** |
| --- | --- | --- |
| chicken β-actin | GTGTGATGGTTGGTATGGGC | 225bp |
|  | CTCTGTTGGCTTTGGGGTTC |  |
| chicken CLCA1 | AGAGGTGGGAGATTGGCATT | 198bp |
|  | GCACCAAGAACAGGCAAGAA |  |
| chicken SLC9A3 | GAGGGAGAGCGTAGAGGTTC | 201bp |
|  | TGTGTCTTCTGTGTCCCGAA |  |
| chicken ALPI | GACCCCAGCGTGACTTATCT | 171bp |
|  | GATCCTCCCACCTTCCACAA |  |
| chicken CD79B | CTCCAGGATCAGCTACGAGG | 221bp |
|  | CTAGGAAGAGGAGCATGGGG |  |
| chicken BLVRA | CCGACTGACTTGGCTGATTG | 169bp |
|  | CTTTTCACGTCTCATCCCCG |  |
| chicken IRF4 | GAGAGAATTTGCCCCGGAAC | 196bp |
|  | ACCAGACCTTATGCTTGGCT |  |
