# Supplementary material for: Transcriptomic Analysis of Gene Expression Patterns in the Cecal Tissue of Liangshan Yanying Chickens and Arbor Acres (AA) Chickens Before 28 Days of Age
Source: Animals (Basel). 2026 Feb 3;16(3):474. doi: 10.3390/ani16030474 (PMC12896458; doi:10.3390/ani16030474)
Supplement: Supplementary file 1 [file animals-16-00474-s001.zip › Supplementary Table S3.docx]

**Supplementary Table S3 Normality Test (Shapiro-Wilk Test) of Cecal Length in Liangshan Yanying Chicken and AA Chicken**

| Breed | Age (d) | Sample Size | Shapiro-Wilk Statistic | P Value | Normality Judgment |
| --- | --- | --- | --- | --- | --- |
| Liangshan Yanying Chicken | 1 | 10 | 0.8905 | 0.1716 | Conforms to normal distribution |
| Liangshan Yanying Chicken | 14 | 10 | 0.8197 | 0.0251 | Does not conform to normal distribution |
| Liangshan Yanying Chicken | 28 | 10 | 0.9358 | 0.5076 | Conforms to normal distribution |
| AA Chicken | 1 | 10 | 0.8365 | 0.0400 | Does not conform to normal distribution |
| AA Chicken | 14 | 10 | 0.7944 | 0.0124 | Does not conform to normal distribution |
| AA Chicken | 28 | 10 | 0.7984 | 0.0139 | Does not conform to normal distribution |

**Note**: Despite normality deviation in some groups, two-way ANOVA demonstrates high robustness, and the equal sample size (n=10 per group) ensures the reliability of the statistical results.
