# Supplementary material for: Transcriptomic Analysis of Gene Expression Patterns in the Cecal Tissue of Liangshan Yanying Chickens and Arbor Acres (AA) Chickens Before 28 Days of Age
Source: Animals (Basel). 2026 Feb 3;16(3):474. doi: 10.3390/ani16030474 (PMC12896458; doi:10.3390/ani16030474)
Supplement: Supplementary file 1 [file animals-16-00474-s001.zip › Supplementary Table S4 .docx]

**Supplementary Table S4 Two-Way Analysis of Variance (Two-Way ANOVA) of Cecal Length in Liangshan Yanying Chicken and AA Chicken of Different Ages**

| Source of Variation | Degrees of Freedom (DF) | Sum of Squares (SS) | Mean Square (MS) | F Value | P Value | Significance |
| --- | --- | --- | --- | --- | --- | --- |
| Breed | 1 | 217.8558 | 217.8558 | 1054.8963 | < 0.001 | *** |
| Age | 2 | 494.6066 | 247.3033 | 1197.4861 | < 0.001 | *** |
| Breed × Age (Interaction Effect) | 2 | 34.5268 | 17.2634 | 83.5925 | < 0.001 | *** |
| Error | 54 | 11.1520 | 0.2065 | - | - | - |
| Total Variation | 59 | 758.1412 | - | - | - | - |

**Note**: **Breed main effect**: F=1054.8963, P<0.001, **extremely significant**, indicating that the cecal length of AA Chickens was significantly longer than that of Liangshan Yanying Chickens at the extremely significant level.

**Age main effect**: F=1197.4861, P<0.001, **extremely significant**, suggesting that cecal length increased significantly with age at the extremely significant level.

**Breed × Age interaction**: F=83.5925, P<0.001, **extremely significant**, implying that the breed difference in cecal length varied with age, and such discrepancy became more pronounced as age increased.

Note: In the above table, *** indicates a highly statistically significant difference.
