# Supplementary material for: Transcriptomic Analysis of Gene Expression Patterns in the Cecal Tissue of Liangshan Yanying Chickens and Arbor Acres (AA) Chickens Before 28 Days of Age
Source: Animals (Basel). 2026 Feb 3;16(3):474. doi: 10.3390/ani16030474 (PMC12896458; doi:10.3390/ani16030474)
Supplement: Supplementary file 1 [file animals-16-00474-s001.zip › Table S2 Optimal Nutrient Levels of Pre-starter and Starter Diets for Broiler Chicks During the Starter Phase.docx]

**Supplementary Table S2 Optimal Nutrient Levels of Pre-starter and Starter Diets for Broiler Chicks During the Starter Phase**

| Nutrient Indicators | Units | Pre-starter Diet (1-14 days of age) | Starter Diet (15-28 days of age) | Standard Basis and Adjustment Notes |
| --- | --- | --- | --- | --- |
| Metabolizable Energy (ME) | MJ/kg (kcal/kg) | 12.54 (3000) | 12.75 (3050) | NY/T 33-2004 Table 4 (baseline value for 0-3 weeks of age); Energy appropriately increased for 15-28 days of age to match the increased feed intake and growth rate of chicks |
| Crude Protein (CP) | % | 21.5 | 21.0 | Adjusted based on the lower limit (21.0%) in NY/T 33-2004 Table 4; Increased by 0.5 percentage points for 1-14 days of age to adapt to the physiological characteristics of immature intestinal tract and low digestive enzyme activity in chicks, ensuring protein supply efficiency |
| Protein-Energy Ratio (CP/ME) | g/MJ | 17.14 | 16.47 | Calculated based on crude protein and metabolizable energy values, meeting the core requirement of energy-nitrogen balance for broiler chicks during the starter phase to avoid protein waste or energy deficiency |
| Calcium (Ca) | % | 1.00 | 0.95 | Fixed value in NY/T 33-2004 Table 4; Slightly decreased for 15-28 days of age to match the skeletal development rhythm of chicks and reduce calcium deposition burden |
| Total Phosphorus (TP) | % | 0.65 | 0.63 | Fixed value in NY/T 33-2004 Table 4; Fine-tuned for 15-28 days of age to form a balanced ratio with available phosphorus |
| Available Phosphorus (AP) | % | 0.45 | 0.43 | Core value in NY/T 33-2004 Table 4; Maintained at a high level due to insufficient phytase secretion in chicks to ensure phosphorus absorption efficiency |
| Lysine (Lys) | % | 1.15 | 1.10 | Based on NY/T 33-2004 Table 4 (1.10%); Increased by 0.05 percentage points for 1-14 days of age. As the first limiting amino acid, it meets the rapid growth needs of chicks |
| Lysine-Energy Ratio (Lys/ME) | g/MJ | 0.917 | 0.863 | Calculated value, conforming to the ratio law of amino acids and energy for broiler chicks during the starter phase |
| Methionine (Met) | % | 0.52 | 0.50 | Fixed value in NY/T 33-2004 Table 4; Slightly increased for 1-14 days of age to ensure the supply of sulfur-containing amino acids and promote intestinal mucosal development |
| Methionine + Cystine (Met+Cys) | % | 0.92 | 0.90 | Fixed value in NY/T 33-2004 Table 4; Slightly increased for 1-14 days of age to meet the developmental needs of immune organs in chicks |
| Threonine (Thr) | % | 0.77 | 0.75 | Fixed value in NY/T 33-2004 Table 4; Slightly increased for 1-14 days of age. As a key amino acid for intestinal mucosal repair, it alleviates intestinal stress in chicks |
| Tryptophan (Trp) | % | 0.23 | 0.22 | Fixed value in NY/T 33-2004 Table 4; Slightly increased for 1-14 days of age to regulate feed intake and neural development of chicks, improving feed intake |
